# Agreement and Reliability of Tinnitus Loudness Matching and Pitch Likeness Rating

**DOI:** 10.1371/journal.pone.0114553

**Published:** 2014-12-05

**Authors:** Derek J. Hoare, Mark Edmondson-Jones, Phillip E. Gander, Deborah A. Hall

**Affiliations:** 1 National Institute for Health Research, Nottingham Hearing Biomedical Research Unit, Nottingham, United Kingdom; 2 Otology and Hearing Group, Division of Clinical Neuroscience, School of Medicine, University of Nottingham, Nottingham, United Kingdom; 3 Department of Neurosurgery, University of Iowa Carver College of Medicine, Iowa City, Iowa, United States of America; University of Texas at Dallas, United States of America

## Abstract

The ability to reproducibly match tinnitus loudness and pitch is important to research and clinical management. Here we examine agreement and reliability of tinnitus loudness matching and pitch likeness ratings when using a computer-based method to measure the tinnitus spectrum and estimate a dominant tinnitus pitch, using tonal or narrowband sounds. Group level data indicated a significant effect of time between test session 1 and 2 for loudness matching, likely procedural or perceptual learning, which needs to be accounted in study design. Pitch likeness rating across multiple frequencies appeared inherently more variable and with no systematic effect of time. Dominant pitch estimates reached a level of clinical acceptability when sessions were spaced two weeks apart. However when dominant tinnitus pitch assessments were separated by three months, acceptable agreement was achieved only for group mean data, not for individual estimates. This has implications for prescription of some sound-based interventions that rely on accurate measures of individual dominant tinnitus pitch.

## Introduction

Tinnitus is characterized by two major components, the phantom percept of sound, and the emotional reaction or perceived threat associated with that sound. Clinical management of tinnitus often aims to address both components. For the percept this can mean either masking or reducing tinnitus awareness by introducing or amplifying external sound, or interrupting tinnitus generating activity in some way. To counter the emotional reaction, education, relaxation, and counseling are used.

Perceptual attributes of tinnitus (pitch, loudness) are recommended as part of patient symptom documentation [1]. Psychoacoustic measures can be used to predict tinnitus malingerers [2], and are essential to the prescription of some sound-based interventions (e.g. [3], [4]. In clinical trials change in psychoacoustic measures of pitch or loudness might be used to interpret a physiological effect of treatments. Hence a standard approach to measuring tinnitus perceptual attributes that demonstrates good agreement and reliability is desirable. Agreement concerns absolute measurement error, i.e. how close repeated measures are to each other, and small measurement error is desirable to detect clinically meaningful change [5]. Reliability concerns how well individual patients or participants can be distinguished from each other based on their data, and despite measurement error [5].

Perceptual attributes such as pitch and loudness are measured by reference to external sounds. However, matching loudness or rating likeness of pitch to a subjective sound is not trivial. Penner et al. [6] and Burns [7] both found that participants more reliably match pitch and loudness when the task involves two external sound sources than for one external and one internalized sound source (their tinnitus). Test procedure can also influence the result. Tyler and Conrad-Armes [8] compared dominant tinnitus pitch match using (1) a ‘method of adjustment’, where the participant was instructed to adjust the pitch of a tone using a dial with wide sweeps to first identify a dominant pitch, using a second dial to fine-tune the selection, (2) a ‘method of limits’, where participants were presented with a sequence of tones ascending or descending at 1/6 octave intervals and asked to rate the likeness as higher or lower, eventually converging on a single ‘higher’ and a ‘lower’ tone which were averaged, and (3) an ‘adaptive method’, similar to the ‘methods of limits’ but where tinnitus pitch was first isolated to within a 1-octave range, and then a ¼ octave range, taking the upper value of the final range to represent the dominant tinnitus pitch. Whilst there was no statistically significant difference in the mean frequency corresponding to dominant tinnitus pitch across the three methods, for some individual's estimates across methods differed considerably with some pitch match differences spanning an octave. Octave confusions were most associated with the ‘method of limits’. In another study, Penner and Saran [9] showed that variability in tinnitus pitch match was significantly greater if participants were allowed to simultaneously adjust the intensity of the matching tones than if the intensity of the matching tones was fixed. In contrast, variability of loudness match did not differ across conditions of fixed or adjustable pitch (i.e. pure tone frequency).

In more recent years, different groups have independently developed automated software tools specifically to use likeness scales and a wide range of frequencies, to measure the psychoacoustic characteristics of human tinnitus. Basille et al. [2] introduced a technique for estimating dominant tinnitus pitch which involves sweeping across pure-tone frequencies to find a match. A ‘slider’ is used to first sweep through all frequencies in the range 0.5 kHz to 20 kHz to identify a frequency similar to the tinnitus pitch, with repeated trial using a narrower and narrower frequency range or ‘brackets’. Noreña et al. [10] used pure tones ranging between 250 Hz and 16 kHz, presented in a random order, to generate a loudness value and tinnitus pitch ‘spectrum’. With this procedure, participants adjust the intensity of pure tones to match the intensity of their tinnitus, and rate on a 0 to 10 numeric scale the extent to which each tonal sound “contributed to the overall tinnitus spectrum”. At the same time, Roberts developed a similar test, the ‘Tinnitus Tester’ [11], [12], which had the option for participants to match or rate sounds that were of different bandwidths (see methods). This automated test is increasingly used in tinnitus research, for example, to compare tinnitus pitch to hearing thresholds [13], to compare cochlear function (hearing thresholds and distortion product otoacoustic emissions) to the tinnitus pitch likeness ratings [14], or to examine the effects of novel interventions on tinnitus loudness or pitch [15], [16]. In a previous validation of his pitch likeness rating and loudness matching procedure, Roberts et al. [12] examined agreement between measures by 17 participants performed at 2–3 week intervals between two sessions. They reported that group mean values for loudness and pitch match did not differ significantly across sessions. This was a limited evaluation as it only involved two sessions and did not account for individual or within-session effects.

The purpose of the present study was to further examine agreement and reliability of this automated approach to estimating tinnitus pitch and loudness in a general research population of people with tinnitus. For two independent datasets, we examined overall levels of absolute agreement between sessions. Whilst acknowledging that tinnitus percept may inherently vary over time for some individuals, we sought to judge reliability using mixed effects modelling to investigate systematic, random, and residual variability in the data.

## Methods

### Ethics statement

Twenty-eight participants were recruited to non-randomised ‘no intervention’ control groups in one of two independent trials (n = 14 in each) which ran concurrently within the same department. For associated publications see Hoare et al. [15] and Davies et al. [17].

Participants were included according to the eligibility criteria of those studies. Ethical approvals of the studies were granted by the National Research Ethics Service for England (Derbyshire and Nottingham Research Ethics Committees). Participants gave their written informed consent to take part in the study in accordance with the approval granted.

### Participants

Baseline characteristics of each group are given in Table 1. Independent sample t-tests showed there to be no significant difference between groups on baseline characteristics (age, hearing loss pure tone average, tinnitus duration, tinnitus handicap, self-reported loudness, sensation level, dominant tinnitus pitch, *p*> 0.05 in all cases).

**Table 1:** Baseline characteristics (mean, SD).

Measure	Group 1	Group 2
Gender	7M, 7F	9M, 5F
Age (years)	53.7 (12.9)	60.9 (8.6)
Pure Tone Average (dB HL)	23 (17)	17 (11)
TI duration (years)	12.6 (15.3)	11.4 (12.8)
Global THQ	966.8 (509.8)	1155.9 (541.2)
Tinnitus loudness VAS (0–100)	46.6 (16.0)	45.6 (21.4)
Tinnitus matched loudness (dB SL)	15 (11)	22 (11)
Frequency with highest likeness rating (kHz)	7.7 (2.9)	6.8 (3.5)

THQ  =  Tinnitus Handicap Questionnaire – range 0–2700, VAS  =  Visual Analogue Scale

All 28 participants were adults with chronic subjective tinnitus (experienced for at least 6 months) who were not receiving any clinical intervention during the study that could affect their hearing or tinnitus. Recruitment was through advertisement on the departmental website and through the department's research volunteer database.

Here, in Study 1 participants performed the Tinnitus Tester assessment five times over 8 weeks at two week intervals (one participant did not complete the fourth and fifth assessment). In Study 2, 14 participants completed the Tinnitus Tester three times over six months at three month intervals.

### Audiometry

Pure-tone audiometry was conducted in a sound-proof booth using a Siemens Unity 2 system and Sennheiser HDA 200 headphones. Pure tone average (PTA) was calculated as the mean threshold for 0.5, 1, 2, and 4 kHz across both ears.

### Tinnitus Tester

Testing was performed in a sound-proofed booth. Participants first completed a 5 minute familiarization procedure that introduced the format of questions (multiple choice, scale bars, adjust loudness level to match ‘x’) and the format of answers (select a response option, select point on scale bar, select volume level). The Tinnitus Tester was then delivered in nine steps, Steps (vi) and (vii) being of primary interest here.

#### (vi) Tinnitus loudness matching

Subjects were presented with 11 sound clips with centre frequencies corresponding to 0.5, 1, 2, 3, 4, 5, 6, 7, 8, 10, and 12 kHz. Bandwidth of these sounds is ‘tonal’ (pure tone), ‘ringing’ (+/−5% characteristic frequency (CF) at −20dB), or ‘hissing’ (+/−15% CF at −20dB) to reflect the tinnitus quality selected earlier. Sounds were presented twice in random order and participants adjusted the loudness of each until it was perceived as equal to that of the tinnitus, up to a maximum of 96 dB SPL with our configuration.

#### (vii) Pitch likeness rating

Participants rated the similarity of the pitch of each of the 11 sound clips to the pitch of their tinnitus on a 100 point likeness scale from 0 (not at all) to 100 (identical). Each sound was presented three times in random order and a profile of the tinnitus frequency spectrum was generated from the average likeness rating for each sound. The frequency with the highest average likeness rating within a session was taken to be the dominant tinnitus pitch.

The other steps required participants to (i) localize their tinnitus sensation to either the left ear, right ear, or both ears, (ii) adjust the loudness of a 0.5 kHz and 5 kHz pure tone to a comfortable sound level, (iii) indicate whether their tinnitus was ‘tonal’, ‘ringing’, or ‘hissing’, (iv) indicate whether tinnitus was steady or pulsing, (v) rate how loud they considered their tinnitus to be on a 100 point visual analogue scale (VAS) with anchors at 0 = “extremely weak” and 100 = “extremely strong”, (viii) perform hearing tests to match the loudness of three masking sounds to the loudness of a 1 kHz tone at 65 dB above threshold, before (ix) performing a test of residual inhibition using those sounds. For the residual inhibition test participants listened to their tinnitus for 30 s, and were then presented with a masker sound for 30 s followed by a silent rating period. During the rating period participants indicated whether they experienced an increase or inhibition of their tinnitus sensation.

### Data analysis

Separate analyses were conducted for each of the 11 test frequencies for both tinnitus loudness matching and pitch likeness rating. To provide better comparability across the timing of the two studies, separate analyses were conducted for Study 1 data where subsets of consecutive sessions were considered (i.e. sessions 1–3, 2–4, 3–5).

Agreement of tinnitus loudness matching, and pitch likeness ratings, was determined as the intraclass correlation coefficients (ICC) of repeated measures over time. This calculates all pairwise correlations whilst avoiding the effect of order. ICC was calculated as a measure of absolute agreement, with pairwise correlations of ≧ 0.7 taken to indicate good agreement [18], and a value of ≧ 0.9 taken as the standard that is required of a tool used for individual and important clinical decision making [19].

Mixed effects modelling techniques were subsequently used to investigate reliability, i.e. sources of significant variability within and across repeated measures. Three parameters were free to vary in the model: (1) Fixed session effects (i.e. the estimated systematic difference across sessions across the whole, not just sampled, population). This gives an indication of any underlying systematic changes from one session to another. A significant fixed session effect would represent a systematic effect over time such as procedural or perceptual learning. (2) Random session effects (within sessions variability, i.e. what between subject variability do we expect to see in the data for a given session). A significant random session effect reflects a ‘consistent variability’ for each participant within each session (e.g. participants may consistently report pitch match values higher or lower during one session, at some or all frequencies). Random session effects therefore give an indication of how well the measure will distinguish between participants across sessions. (3) Error variance. This corresponds to the residual variability where significance implies there is variation within the measure that you should expect to see between the results of a single subject over sessions. Sources of error variance commonly relate to test construction, administration, or methods of scoring and interpretation.

ICC analyses of loudness and pitch across all frequencies, and for the dominant pitch, were conducted in SPSS (Version 20). For each participant, octave differences in dominant tinnitus pitch were also calculated, comparing the dominant tinnitus pitch in Session 1 with all subsequent sessions. Octave differences across sessions were compared in a repeated-measures ANOVA. Mixed-effects modelling was conducted in R (version 3.0.0).

For one participant, the second measure was lost due to a corrupted data file. The modelling approach selected permitted available data from participants with some missing responses to be included. Missing data were not imputed therefore, but were assumed to be missing completely at random. Significance was taken as *p* <0.05.

## Results and Discussion

### Participant and qualitative tinnitus characteristics

Of 28 participants, 20 consistently reported that their tinnitus was localized to either their left ear (n = 6), both ears (n = 11) or their right ear (n = 3) on each assessment visit. The remaining eight participants reported a shift in their tinnitus location on at least one occasion, either a change from perceiving tinnitus in the left or right ear to both, or a change from perceiving tinnitus in both ears to either the left or the right ear. In terms of bandwidth according to the sound clip examples, seven participants consistently indicated their tinnitus was ‘hissing’, none consistently chose ‘ringing’ and 12 consistently indicated their tinnitus was tonal. The remaining nine participants indicated that the spectral qualities of their tinnitus changed between at least two of the three options at different time points. Twenty-five participants consistently reported a steady tinnitus sound and none consistently reported experiencing a pulsing sound. The other three participants reported that their tinnitus was steady or pulsing on different occasions. Fifteen of 28 participants consistently reported a reduction in tinnitus loudness during the residual inhibition test. The reduction was however similar across all sound stimuli types.

### Loudness matching and pitch likeness rating

**Figure 1:**
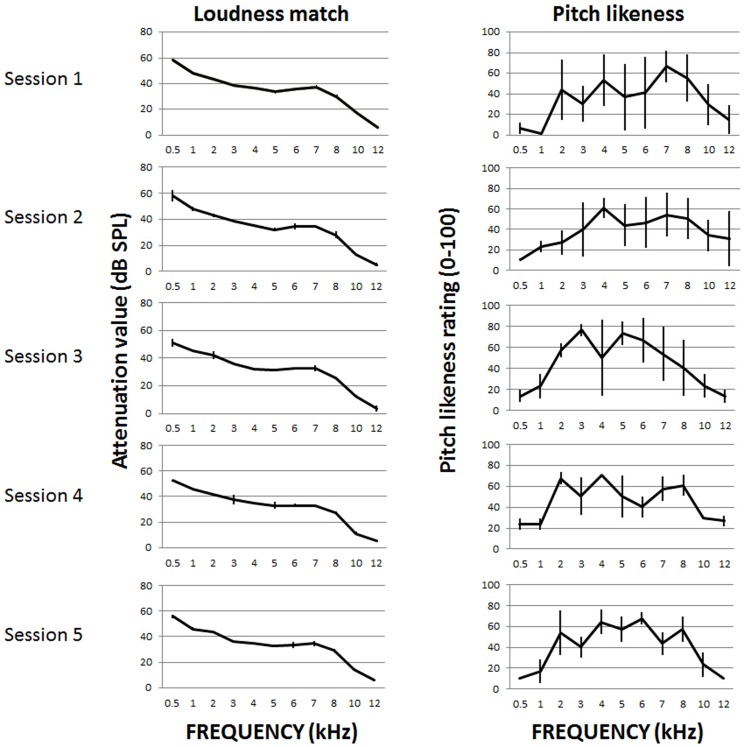
Example data from a Study 1 participant with tonal tinnitus. In Step 3 of the ‘Tinnitus Tester’ participant always selected ‘Tonal’. Data are presented as mean ± SD for each test frequency. Loudness match is reported as the PA5 attenuator value, 0 attenuation (maximum output of the system) was 96 dB SPL.

**Figure 2:**
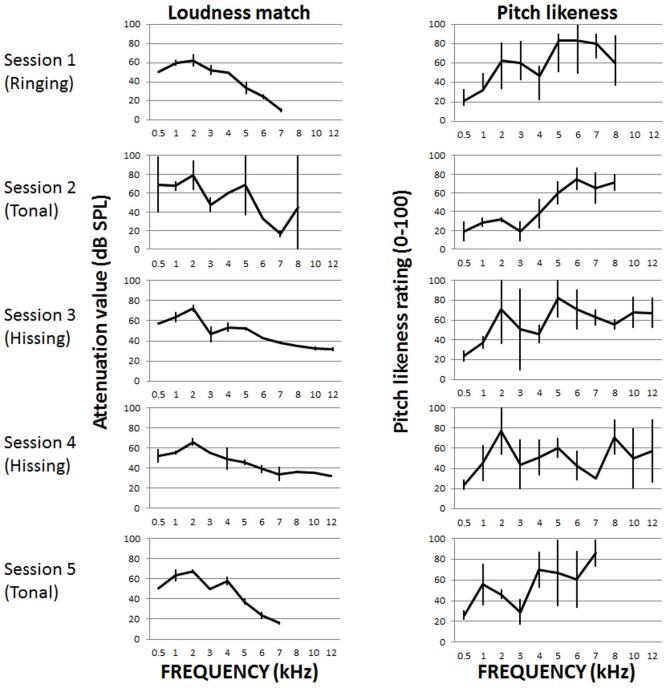
Example data from a Study 1 participant with ‘atonal’ tinnitus. In Step 3 of the ‘Tinnitus Tester’ participant selected different bandwidths across sessions (selection in brackets). Data are presented as mean ± SD for each test frequency. Loudness match is reported as the PA5 attenuator value, 0 attenuation (maximum output of the system) was 96 dB SPL.

Figures 1 and 2 showcase data to demonstrate the types of variability observed across and between the data for individual participants. Figure 1 shows data from a participant who consistently selected ‘Tonal’ in Step 3. In this example, the pattern of loudness matching data is particularly well conserved across sessions, with very little deviation from mean values in any case. In contrast, the pattern or ‘spectrum’ generated in the pitch matching exercise shows less consistency. For individual frequencies the standard deviation of the pitch estimate is reduced in later sessions possibly due to some learning taking place. Moreover, few of the spectra represent tonal tinnitus. In particular, in Sessions 4 and 5 the spectrum is quite flat. This may represent an inability to perform the procedure or actual changes in the components of the individual's tinnitus over time.

Figure 2 shows data from a participant who selected different bandwidth options on different occasions. So either the sound quality of the participant's tinnitus has naturally fluctuated over time, or none of the options were sufficiently similar and the participant selected a random option. Here, in Session 1 loudness matching to ‘ringing’ sounds was associated with little variability, but in Session 2 where the ‘tonal’ option was selected in Step 3, there were substantial standard deviations for matching at a number of frequencies. Variability in loudness matching was reduced again in Sessions 3 and 4 (hissing), and remained low in Session 5 even though ‘tonal’ sounds are used. Again, low standard deviations on individual frequencies may be the result of learning. Variability both within and across sessions was also observed in pitch matching. Of particular note, sounds with higher centre frequencies (10 and 12 kHz) were only heard by this participant when the ‘hissing’ option was selected.

### Agreement

**Figure 3:**
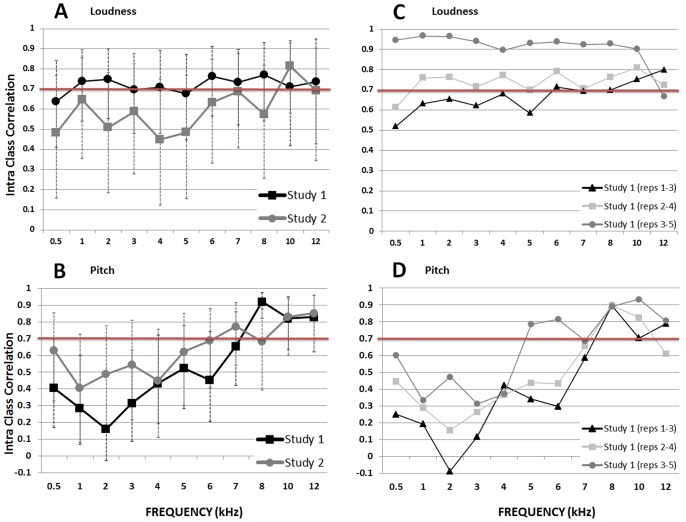
Tinnitus loudness matching and pitch likeness rating. Intraclass Correlation Coefficient (ICC) values for (A) loudness matching, and (B) pitch likeness, in Study 1 (5 repeated measures) and Study 2 (3 repeated measures), ± 95% CI. ICC values for Study 1 grouped according to three consecutive sessions for (C) loudness matching, and (D) pitch likeness. ICC> 0.7 (above red line) is considered good for group data and ICC> 0.9 is good for individual patient data.

Average ICCs (± 95% CI) for tinnitus loudness matching at each test frequency across all sessions are given in Figure 3. ICC values > 0.7 are taken as acceptable agreement to interpret group level data. ICC here shows the level of absolute agreement across test sessions on this measure. In Study 1, the average ICC was ≧ 0.7 for loudness match at all frequencies except for 0.5 (ICC = 0.64, CI = 0.41–0.84) and 5 kHz (0.68, CI = 0.45–0.87) (Figure 3A). In Study 2, average ICC exceeded 0.7 for loudness match at only 10 kHz (ICC = 0.81, CI = 0.58–0.94). ICC values > 0.9 are taken as acceptable agreement to interpret individual participant data. In both studies, ICCs for all frequencies were below this value.

Average ICCs for pitch likeness rating are given in Figure 3B. In contrast to loudness match data, the average ICC value for pitch likeness ratings was ≧ 0.7 at only a few high frequencies in each study (8, 10, and 12 kHz in Study 1, and 7, 10, and 12 kHz in Study 2) (Figure 3B). Agreement on pitch-likeness rating exceeded 0.9 at only 8 kHz.

Sub-analyses of Study 1 loudness data by three consecutive sessions showed a consistent increase in average ICC at most frequencies (Figure 3C). Notably, average ICCs across Sessions 1–3 were <0.7 at seven of the 11 test frequencies. However by Sessions 2–4, average ICCs were > 0.7 at all frequencies except 0.5 kHz. Across Sessions 3–5, average ICC were ≧ 0.9 at all test frequencies except 12 kHz. Although overall for Study 1, the ICC ranged quite consistently from 0.7 to 0.8 across frequencies (Figure 3A), there is evidence that some learning of the procedure occurred over time. Notably, ICC ranges from 0.9 to 1 were achieved across frequencies if the initial Sessions 1 and 2 were omitted from the analysis.

A further post hoc analysis was conducted on data collected in Step 5 where loudness was self-rated on a 100-point VAS. As well as providing a comparison of reliability with the loudness matching procedure used in Step 6, this also allowed us to consider how much variability in the data might be associated with either natural fluctuations in tinnitus or with the ‘Tinnitus Tester’ procedure itself. As with the loudness matching, within subject variability was observed for all participants. Across assessments differences on the 100-point VAS varied from as little as 1 point for one participant, to as much as 61 points for another (Table S1). However, absolute agreement was higher for the VAS self-reported loudness than for the matched tinnitus loudness. In Study 1 the ICC for VAS self-reported loudness was 0.93 (CI = 0.85–0.98), in Study 2 it was 0.86 (CI = 0.66–0.95). This indicates that variability in matched loudness is unlikely to be wholly attributable to fluctuations in the tinnitus percept over time.

For pitch likeness rating sub-analyses of Study 1 data (Figure 3D) by three consecutive sessions showed that ICC values reached ≧ 0.7 for three frequencies across Sessions 1–3, just two frequencies across Sessions 2–4, and five test frequencies across Sessions 3–5. This may again suggest some learning during earlier sessions. Strongest agreement (ICC up to ∼ 0.9) was seen at higher frequencies (likely corresponding to the dominant tinnitus pitch) and participants’ weakest agreement was at lower frequencies which were generally least similar to the tinnitus pitch. For frequencies between 0.5 and 4 kHz, absolute agreement increased only slightly if earlier sessions were ignored, but never reached > 0.7.

Further analyses were conducted to measure the ICC of dominant tinnitus pitch estimates across sessions. Dominant pitch was taken as the frequency which was given the greatest numerical likeness rating. In Study 1, none of the participant showed an absolute agreement across the five sessions, although overall ICC was high (0.90, CI = 0.8–0.97), just reaching a level acceptable for individual clinical decision making. Inter-item correlations showed no obvious order effect. For example, correlations were strongest between Sessions 1 and 3 (*r* = 0.91), and Sessions 3 and 4 (*r* = 0.94), whereas the correlation between Sessions 4 and 5 was weak (*r* = 0.56). Differences in dominant tinnitus pitch between baseline and Sessions 2 to 5 ranged from a difference of 0 to a difference of 1.56 octaves. For four of the 14 participants in Study 1, the difference across sessions exceeded one octave on at least one occasion. The effect of time was not significant (*F* = 1.136 (2.085,25.025), *MSE* = 0.385, *p* = 0.339).

In Study 2, two participants showed absolute agreement across the three sessions, i.e. they selected the same dominant pitch on every occasion. However, for these two cases the dominant pitch was 0.5 kHz and 12 kHz, at the two extremes of the test range. Overall ICC was good although lower than in Study 1 (0.85, CI = 0.61–0.95), so acceptable for interpreting group level data but insufficient for individual clinical decision making. Inter-item correlation increased from 0.55 between Sessions 1 and 2, to 0.85 between Sessions 2 and 3. Differences in dominant tinnitus pitch between baseline and Sessions 2 and 3 ranged from a difference of 0 to a difference of 4.3 octaves (one participant rated their dominant tinnitus pitch as 10, 0.5 then 1 kHz across the three sessions). For just one other participant, estimates varied across sessions by ≥ 1 octave. Again the effect of time showed no obvious pattern and was not significant overall (*F* = 0.283 (2,24), *MSE*  = .197, *p* = 0.756).

### Reliability

Outputs from the mixed effects modelling analyses are provided in Tables 2 and 3.

**Table 2:** Model output for tinnitus loudness matching.

Fixed Effect	Study 1						Study 2			
	Session 1	Session 2	Session 3	Session 4	Session 5	p value	Session 1	Session 2	Session 3	p.value
500 Hz	61.89	50	51.79	53.78	52.89	***0.00246***	60.18	63.88	54.89	***0.00156***
1 kHz	63.82	51.25	54.79	53.75	53.85	***0.04890***	63.43	66.77	60.82	***0.00314***
2 kHz	62.54	49.43	52.14	52.63	52.04	***0.02533***	62.79	67.06	58	***0.00001***
3 kHz	56.04	42.86	45.04	48.06	45.87	***0.00700***	57.14	60.28	53.64	***0.01519***
4 kHz	48.64	38.3	43.64	42.24	42.86	0.05693	49.86	54.97	46.61	***0.01859***
5 kHz	40.42	33.93	35.71	37.59	35.63	0.57683	44.14	51.68	40.29	***0.00316***
6 kHz	37.05	31.28	34.36	34.48	33.01	0.74008	40.43	46.6	38.96	0.15076
7 kHz	33.36	26.59	32.05	32.47	29.99	0.52453	39.07	47.4	37.11	0.08560
8 kHz	34.67	25.2	29.64	28.86	26.3	0.25369	33.14	43.35	34.11	0.22396
10 kHz	28.08	26.78	22.03	23.7	22.45	***0.00234***	33.8	38.76	30.2	0.03990
12 kHz	23.2	17.25	21.49	19.99	23.48	0.23892	23.42	30.09	22.83	0.25492

Random Effects SD	Study 1						Study 2			
	Session 1	Session 2	Session 3	Session 4	Session 5	p value	Session 1	Session 2	Session 3	p.value
500 Hz	12.62	24.17	17.62	16.28	19.09	***0.00055***	13.18	12.25	13.15	***0.00575***
1 kHz	20.88	27.06	21.17	22.63	23.43	***<0.00001***	11.85	12.66	11.72	***0.02898***
2 kHz	20.97	27.58	21.38	21.67	22.47	***0.00001***	11.85	11.52	12.43	***0.00227***
3 kHz	19.19	24.64	20.24	20.02	19.37	***<0.00001***	10.71	13.34	12.29	0.11390
4 kHz	20.22	25.69	21.17	20.53	21.75	***<0.00001***	10.5	18.62	12.7	***0.00002***
5 kHz	20.08	26.46	20.26	18.7	19.17	***0.00001***	11.72	21.55	14.5	***<0.00001***
6 kHz	20.85	23.4	20.89	20.41	20.54	***<0.00001***	16.68	16.38	17.12	0.21550
7 kHz	22.75	22.65	19.2	17.01	19.04	***<0.00001***	16.23	19.58	17.33	***0.00039***
8 kHz	22.67	19.83	18.99	18.85	19.54	***<0.00001***	17.93	20.56	17.89	***<0.00001***
10 kHz	17.11	15.48	17.44	17.45	16.69	0.07578	17.28	26.51	17.23	***<0.00001***
12 kHz	16.55	17.54	17.1	17.1	19.29	***0.00008***	16.05	24.42	14.24	0.11539

Error SD	Study 1						Study 2			
	Session 1	Session 2	Session 3	Session 4	Session 5	p value	Session 1	Session 2	Session 3	p.value
500 Hz	9.37	9.45	6.59	5.63	5.8	***<0.00001***	3.56	6.1	4.5	***0.00051***
1 kHz	2.56	3.05	3.29	2.45	3	0.13957	5.61	4.7	4.6	0.21539
2 kHz	6.86	6.32	6.02	4.8	5.03	***0.02360***	7.29	5.79	5.6	0.07558
3 kHz	3.1	3.51	4.02	4.51	4.69	0.01067	6.88	6.17	6.84	0.66663
4 kHz	2.47	2.71	4.94	3.26	3.29	***<0.00001***	5.16	4.58	4.08	0.21716
5 kHz	2.97	5.37	3.07	4.05	4.3	***0.00004***	3.7	3.78	3.82	0.97161
6 kHz	1.85	2.95	2.78	3.4	3.74	***0.00011***	6.26	9.05	4.41	***<0.00001***
7 kHz	2.52	3.43	3.33	2.7	1.84	***0.00018***	5.61	6.63	6.36	0.43430
8 kHz	3.61	7.56	2.95	3	2.91	***<0.00001***	5.79	6.63	5.23	0.21419
10 kHz	4.06	3.59	3.81	3.81	8.91	***<0.00001***	6.88	4.28	4.76	***0.00050***
12 kHz	2.11	2.24	2.44	3.3	3.95	***0.00011***	2.91	6.22	4.89	***0.00005***

Bold italic indicates the type of variability that was significant (uncorrected) for that frequency within each study.

**Table 3:** Model output for tinnitus pitch likeness rating.

Fixed Effect	Study 1						Study 2			
	Session 1	Session 2	Session 3	Session 4	Session 5	p value	Session 1	Session 2	Session 3	p.value
500 Hz	19.64	11.14	11.83	19.14	13.73	0.23783	17.71	23.49	27.81	***0.03917***
1 kHz	29.26	16.31	23.98	27.37	27.37	0.30696	24.36	28.9	32.29	0.27718
2 kHz	31.74	33.81	39.67	36.25	38.55	0.73316	30.17	35.59	32.36	0.49239
3 kHz	42.64	45.86	41.76	48	41.23	0.46369	28.52	41.32	38.64	0.06064
4 kHz	49	53.14	48.05	42.21	51.52	0.30390	41.26	50.77	46.62	0.36415
5 kHz	53.52	64.24	49.64	51.98	49.15	0.14563	48.5	49.49	45.57	0.57956
6 kHz	63.95	65.39	59.61	56.07	54.48	0.60314	45.36	55.32	48.98	0.13639
7 kHz	64.86	62.58	52.22	54.57	58.16	0.27504	48.29	59.28	53	***0.02243***
8 kHz	63.26	59.46	60.7	57.44	59.3	0.75535	54.79	55.02	55.5	0.99174
10 kHz	61.4	55.09	64.34	57.73	62.8	0.08052	55.07	57.55	50.86	0.21236
12 kHz	61.92	63.54	55.06	57.22	61	0.88275	45.2	50.44	43.66	0.29350

Random Effects SD	Study 1						Study 2			
	Session 1	Session 2	Session 3	Session 4	Session 5	p value	Session 1	Session 2	Session 3	p.value
500 Hz	19.81	12.36	11.24	16.82	13.14	***<0.00001***	14.18	21.8	19.01	***0.00023***
1 kHz	20.78	15.04	15.74	10.23	10.04	***0.00062***	15.08	17.95	13.91	***0.00039***
2 kHz	18.71	21.06	16.14	19.04	11.24	***<0.00001***	16.74	16.75	14.22	0.07242
3 kHz	15.11	15.08	10.45	17.64	15.53	0.55665	19.46	21.98	17.7	***0.00849***
4 kHz	11.86	18.61	18.83	14.17	10.48	0.31899	23.06	26.33	15.95	***0.00069***
5 kHz	15.54	15.12	23.02	28.09	23.37	***0.00001***	20.74	21.74	19.24	0.14328
6 kHz	13.3	18.25	21.64	20.12	22.25	***0.01155***	22.36	23.15	23.08	0.16778
7 kHz	19.75	25.58	27	30.07	26.2	***<0.00001***	26.43	26.53	26.12	***0.00436***
8 kHz	21.27	27.15	24.81	30.99	29.54	0.55930	23.82	28.75	24.03	***0.00348***
10 kHz	23.13	27.32	27.61	34.15	34.68	***0.01347***	26.31	29.43	28.54	***0.04378***
12 kHz	15.66	27.42	32.23	29.64	30.9	***<0.00001***	26.65	29.33	29.02	0.74245

Error SD	Study 1						Study 2			
	Session 1	Session 2	Session 3	Session 4	Session 5	p value	Session 1	Session 2	Session 3	p.value
500 Hz	14.51	12.72	10.09	10.76	11.92	***0.00076***	10.15	12.74	9.35	***0.00303***
1 kHz	17.2	12.99	16.4	17.06	18.25	***0.01134***	15.65	11.4	12.95	***0.00406***
2 kHz	13.95	12.38	16.08	15	15.83	***0.04664***	16.32	16.47	13.39	0.06437
3 kHz	20.46	21.4	23.37	17.73	17.71	***0.00552***	15.13	16.97	12.51	***0.00828***
4 kHz	19.52	15.79	18.37	20.33	20.47	0.05360	14.71	15.14	15.78	0.75703
5 kHz	19.24	18.74	15.71	15.51	17.77	0.05046	15.36	16.71	13.71	0.12533
6 kHz	20.53	18.81	16.8	19.76	20.47	0.19689	14.82	17.42	15.28	0.19154
7 kHz	15.48	15.23	13.94	12.06	13.77	0.07778	14.62	16.37	12.76	***0.03759***
8 kHz	17.38	14.77	14.73	14.47	15.47	0.13913	17.46	17.93	14	***0.02737***
10 kHz	16.61	17.02	13.97	13.87	10.76	***0.00012***	11.6	13.95	11.69	0.09947
12 kHz	23.38	14.86	15.69	16.21	12.83	***<0.00001***	16.48	16.63	13.49	0.08268

Bold italic indicates the type of variability that was significant (uncorrected) for that frequency within each study.

#### Loudness - fixed effects

In Study 1, fixed effects for loudness matching were higher in Session 1 than any other, for all frequencies except 12 kHz (Table 2). This effect was most pronounced (significant) for the lower frequencies (0.5 to 3 kHz) and for 10 kHz. For Study 2, loudness match values in Session 2 were higher than Sessions 1 or 3 and this effect reached statistical significance for lower frequencies (0.5 to 5 kHz). The differences observed between the two studies in terms of the fixed effects may be accounted for by the differences in duration of the session intervals. The intersession interval in Study 2 was three months, compared to just two weeks in Study 1, so conceivably procedural or perceptual learning effects would be less pronounced or indeed absent.

#### Loudness - random effects

In Study 1, the random effect variance was typically highest at Session 2 and statistically significant for all frequencies *except* 10 kHz. Study 2 was consistent in showing higher random effect variance in Session 2 than Sessions 1 or 3.

#### Loudness - error variance

In Study 1 error variance was low in Session 1, higher in Session 2, and gradually reduced across Sessions 3–5. This could be interpreted as variability in the systematic effect that was typically observed between the first and second sessions. In Study 2 random effect variance was higher in Session 2 than Sessions 1 or 3.

The consistency of random and error variance across the two studies may be coincidental or reflect some (cognitive) change in the approach taken by participants in their second experience with the test. It may also reflect differences in the degree of ‘learning effect’ between individuals. In either case it would suggest that if researchers wish to minimize variability in within-session data or to consider individual case studies for example, then two familiarization sessions are desirable.

#### Pitch – fixed effects

For tinnitus pitch likeness ratings, the main fixed effect was largely non-significant for both studies, i.e. the level of agreement for likeness rating for most test frequencies was consistent across time (Table 3). The only exceptions were in Study 2 where mean likeness rating for 0.5 kHz increased with session, and 7 kHz where mean rating peaked in Session 2, but these do not point to there being a significant fixed effect on the performance of pitch matching.

#### Pitch – random effects

Random effects on pitch likeness ratings in Study 1 were less apparent in Session 1 and more pronounced in later sessions, particularly at higher frequencies. For 8 out of 11 frequencies the differences across sessions was statistically significant indicating difference in reliability between sessions. In Study 2, variability due to random effects was smaller in Sessions 1 and 3 than in Session 2 and overall was statistically significant for 7 out of 11 frequencies.

#### Pitch – error variance

In Study 1 error variance was significant for 6 out of 11 frequencies with most seen in Session 1, and least in Sessions 3 and 4. There are two possible explanations for this variance. First, there may have been differences in learning between individuals. At Session 1 all participants were psychophysical ‘novices’, but with practice some individuals were able to likeness-rate pitch consistently, whereas others were not [20]. Second, for some participants the tinnitus sound may not have been stable from session to session. In Study 2 error variance was significant for 5 out of 11 frequencies with most seen in Session 2, and least in Session 3.

Overall, and although less reliable than tinnitus loudness matching, degree of reliability of pitch likeness rating across multiple frequencies appeared consistent over time, and there was no predictable relationship between session number and variability within a session or within trials.

## Conclusions

Here we examined agreement and reliability of tinnitus pitch and loudness estimates in the control groups of two independent studies where participants completed either three or five repeats of the Tinnitus Tester, over 6 months or 8 weeks respectively. Participants were selected according to the specific requirement of the individual studies. As such, the analysis here represents validation of the Tinnitus Tester for a general tinnitus trial population. It is possible that with a more selected sample, a group of participants who self-report a constant tonal tinnitus for example, we might observe higher between- and within-session agreement. However, this speculation requires empirical evaluation.

Our interpretation of the findings is that in a repeated measures design, the loudness matching procedure is subject to early practice effects and for confidence in group level data at least one assessment should be conducted to overcome learning effects, taking the second assessment as baseline. This effect is evident elsewhere in the acoustic literature. For example, Brännström et al. [21] concluded that order effects influence the average acceptable noise level (ANL). They tested repeatability over four sessions of the ANL test, using diotic presentation and normal-hearing listeners and observed a two-fold difference in the coefficient of reliability across sessions. Agreement between Sessions 3 and 4 was greatly increased compared to agreement between Sessions 1 and 2. Given the fixed effect on loudness matching observed here we consider it important to factor the effect when developing protocols for clinical trials for example. Landgrebe et al. [22] outlines a proposal for an international standard for the conduct of clinical trials on tinnitus which accounts the need to establish a stable baseline of tinnitus severity. It is recommended that a tinnitus severity questionnaire is administered a number of times pre-intervention with the average being taken to be a stable baseline. In the case of psychoacoustic measures of tinnitus loudness however we would recommend that a more suitable approach is to disregard at least the initial measure, taking the second or later pre-intervention measure as a baseline. In clinic, wherever loudness matching is to be used to inform important clinical decisions (requiring ICC of > 0.9 for confidence) then it would be prudent to disregard the first two loudness match assessments and select the third loudness match as a baseline. In practice, sessions may need to be in very close succession so as not to delay treatment. Loudness matching is most reliable after two assessments and reaches agreement levels that are clinically acceptable. Our results support the use of loudness matching as reliable for the diagnostic workup of tinnitus patients, and as a potential tool to identify tinnitus malingerers as suggested by Basile et al. [2].

Here pitch likeness rating across a range of frequencies appeared an inherently more difficult task than loudness matching and absolute agreement never reached a clinically acceptable level. Agreement was poorest at lower frequencies (for frequencies up to 4 kHz agreement never reached a value considered necessary for confidence) possibly due to a lack of confidence in rating likeness when the dominant tinnitus pitch is typically high frequency. Learning effects have been clearly noted in previous studies when participants were trained on auditory tasks such as frequency discrimination training [23], [24] or even where participants were repeatedly exposed to sound stimuli in an impossible discrimination task [25]. Reliability assessments for pitch matching across a range of frequencies suggest it may be a factor for some individuals but in general variability in pitch likeness ratings across multiple frequencies was high and unpredictable. Estimates of dominant tinnitus pitch were more consistent however. Good agreement on dominant tinnitus pitch in Study 1 supports individual clinical decision-making, but not so the result for Study 2, pointing to an effect of the duration between sessions. Thus, our data provide mixed support for using dominant tinnitus pitch to reliably evaluate changes in tinnitus pitch or to accurately prescribe individualized sound therapy such as acoustic coordinated reset neuromodulation [4] or notched noise [26]. Prescription strategies may have some tolerance for the observed variance in pitch matching, but for important clinical decision making, acceptable tolerance limits should be clearly established and stated as part of the patient assessment and therapy procedure. In particular, an individual participant for whom the dominant pitch estimate exceeds one octave across sessions may not be a good candidate for such therapeutic approaches as the reliability of the matching procedure is questionable.

## Supporting Information

Table S1 **Loudness ratings on a 100 point visual analogue scale.**
(DOCX)
