# Supplementary material for: Agreement and Reliability of Tinnitus Loudness Matching and Pitch Likeness Rating
Source: PLoS One. 2014 Dec 5;9(12):e114553. doi: 10.1371/journal.pone.0114553 (PMC4257689; doi:10.1371/journal.pone.0114553)
Supplement: Table S1 — Loudness ratings on a 100 point visual analogue scale. (DOCX) [file pone.0114553.s001.docx]

Table S1.

| **Study 1** | **T1** | **T2** | **T3** | **T4** | **T5** |
| --- | --- | --- | --- | --- | --- |
| Participant 1 | 41 | 30 | 74 | 40 | 42 |
| Participant 2 | 21 | 16 | 10 | 21 | 22 |
| Participant 3 | 59 | 51 | 41 | 61 | 35 |
| Participant 4 | 25 | 30 | 25 | 30 | 30 |
| Participant 5 | 91 | 30 | 50 | 61 | 30 |
| Participant 6 | 52 | 95 | 89 | 91 | 93 |
| Participant 7 | 30 | 27 | 40 | 27 | 24 |
| Participant 8 | 18 | 14 | 26 | 12 | 12 |
| Participant 9 | 35 | 46 | 35 | 35 | 30 |
| Participant 10 | 71 | 54 | 68 | 62 | 70 |
| Participant 11 | 69 | 79 | 88 | missing | |
| Participant 12 | 31 | 30 | 26 | 30 | 29 |
| Participant 13 | 55 | 56 | 56 | 63 | 68 |
| Participant 14 | 41 | 61 | 61 | 61 | 71 |
|  | | | | | |
| **Study 2** | **T1** | **T2** | **T3** |  | |
| Participant 1 | 71 | 89 | 68 |  |  |
| Participant 2 | 68 | 32 | 30 |  |  |
| Participant 3 | 36 | 35 | 31 |  |  |
| Participant 4 | 30 | 31 | 30 |  |  |
| Participant 5 | 51 | 81 | 69 |  |  |
| Participant 6 | 70 | 71 | 61 |  |  |
| Participant 7 | 38 | 41 | 50 |  |  |
| Participant 8 | 50 | 36 | 45 |  |  |
| Participant 9 | 26 | 25 | 40 |  |  |
| Participant 10 | 30 | 20 | 41 |  |  |
| Participant 11 | 30 | 28 | 30 |  |  |
| Participant 12 | 41 | 41 | 61 |  |  |
| Participant 13 | 50 | 19 | 29 |  |  |
| Participant 14 | 61 | 61 | 71 |  |  |

Loudness ratings on a 100 point visual analogue scale
